# Short-term fasting early in life extends lifespan in a mite *Amblydromalus limonicus* (Acari: Phytoseiidae)

**DOI:** 10.1038/s41598-025-12152-x

**Published:** 2025-07-20

**Authors:** Pei-Ying Peng, Zhi-Qiang Zhang

**Affiliations:** 1 Institute of Microbiology, Qujing Medical College, Qujing, 655100 Yunnan Province China; 2 Manaaki Whenua Landcare Research Group, Bioeconomy Science Institute, 231 Morrin Road, Auckland, 1072 New Zealand; 3 School of Biological Sciences, The University of Auckland, 231 Morrin Road, Auckland, New Zealand

**Keywords:** Caloric restriction, Intermittent fasting, Aging, Biological control, Life duration, Ecology, Zoology

## Abstract

Dietary restriction is one of the most effective and reproducible dietary interventions known to regulate aging and increase the healthy lifespan in various model organisms, ranging from the unicellular yeast to worms, flies, rodents, and primates. This study examined the effects of short-term fasting during early life (STFEL) on longevity in the phytoseiid predatory mite *Amblydromalus limonicus*, hypothesizing that STFEL—especially at the facultative feeding larval stage—would extend lifespan. Experimental mites were divided into a control group with no fasting and four treatment groups subjected to 1-day fasting at the start in different developmental stages: Larval (T1), protonymph (T2), deutonymph (T3), and adult (T4). Results demonstrate that STFEL could significantly extends lifespan, with the most pronounced effects observed when fasting occurred at the start of the larval stage compared to other periods. These findings highlight the adaptive role of facultative feeding larvae and provide a foundation for developing physiological enhancement strategies in biocontrol applications.

## Introduction

Dietary restriction (DR) is also called calorie restriction (CR), and refers to a reduction in food intake without malnutrition. According to resource allocation theory, animals face a trade-off between the allocation of resources into reproduction and into individual growth/maintenance^1–3^. This trade-off is reinforced when food conditions decline^4,5^. It is well established in gerontological research that many animals increase their lifespan when food is in suboptimal supply for growth or reproduction. It was first noted in the 1930s that food restriction significantly extends the lifespan of rodents^6^. DR extends lifespan in a remarkable range of organisms, including yeast, rotifers, spiders, worms, fish, mice, and rats^7^.

Studies on arthropods have demonstrated various effects of fasting and starvation : (1) prolonged developmental periods, reduced survival rates, and impaired fecundity (e.g., smaller offspring and lower egg viability); (2) species-specific starvation tolerance, such as the gradual decline in survival of apple woolly aphids versus precocious pupation in fall webworm larvae under starvation; (3) behavioral adaptations like migration, increased foraging activity, and neural regulation of feeding (e.g., AstA-NPF pathway); (4) contextual benefits, such as enhanced predatory capacity in natural enemies (e.g., *Arma chinensis*) after short-term fasting, improving biocontrol efficacy. These findings highlight the multifaceted strategies arthropods employ to cope with food scarcity^8–11^.

Emerging data show that its effect may also apply to non-human primates^12^. Additionally, DR can decrease the incidence and progression of many age-dependent diseases, including cardiovascular and cerebrovascular systems, cancer, Alzheimer’s disease and diabetes^13–16^. Dietary restriction can also prevent age-associated declines in psychomotor and spatial memory tasks in humans, as well as the loss of dendritic spines necessary for learning and improves the brain’s plasticity and ability for self-repair^17–19^. However, beneficial effects in a mouse model for amyotrophic lateral sclerosis were not observed^20^.

The concept of DR has been expanded from initial calorie restriction (CR) to a wide range of diet-related interventions, including short-term starvation, intermittent fasting, and macronutrient restriction^4^. Intermittent fasting (IF; diets with reduced meal frequencies such as every-other-day-fasting) can also increase lifespan, even when there is little or no overall decrease in calorie intake^9,21^. An increasing number of physiological effects of CR and IF that may contribute to their abilities to increase lifespan have been documented in studies on rodents, monkeys, and humans^22,23^.

In our study, we used a phytoseiid predatory mite—*Amblydromalus limonicus* (Garman and McGregor)—to study the relationship between one-time (1 day) short-term fasting during early life (STFEL) and lifespan. We hypothesized that STFEL—especially in the facultative feeding larva—could extend the lifespan. This is a topic that has not been previously explored for most model animals. There is also limited information about the relationship between DR and lifespan of *A. limonicus*, one of the most important biocontrol agents for greenhouse pests^24^. The species was described in 1956 from citrus in California; its distribution range covers North and South America, Australia and New Zealand. It first caught the attention of biocontrol workers in the 1960s as a natural enemy of the spider mites (e.g. *Oligonychus punicae* (Hirst)) in avocados and other fruit trees. It is a generalist predatory mite with economic potential to control thrips and whiteflies in protected cultivation^24–27^. Hence, this species also has great applied importance. As the first systematic exploration of early-life dietary interventions in predatory arthropods, our work lays groundwork for developing physiological enhancement strategies in biocontrol applications.

## Results

### Immature survival and development

**Fig. 1:**
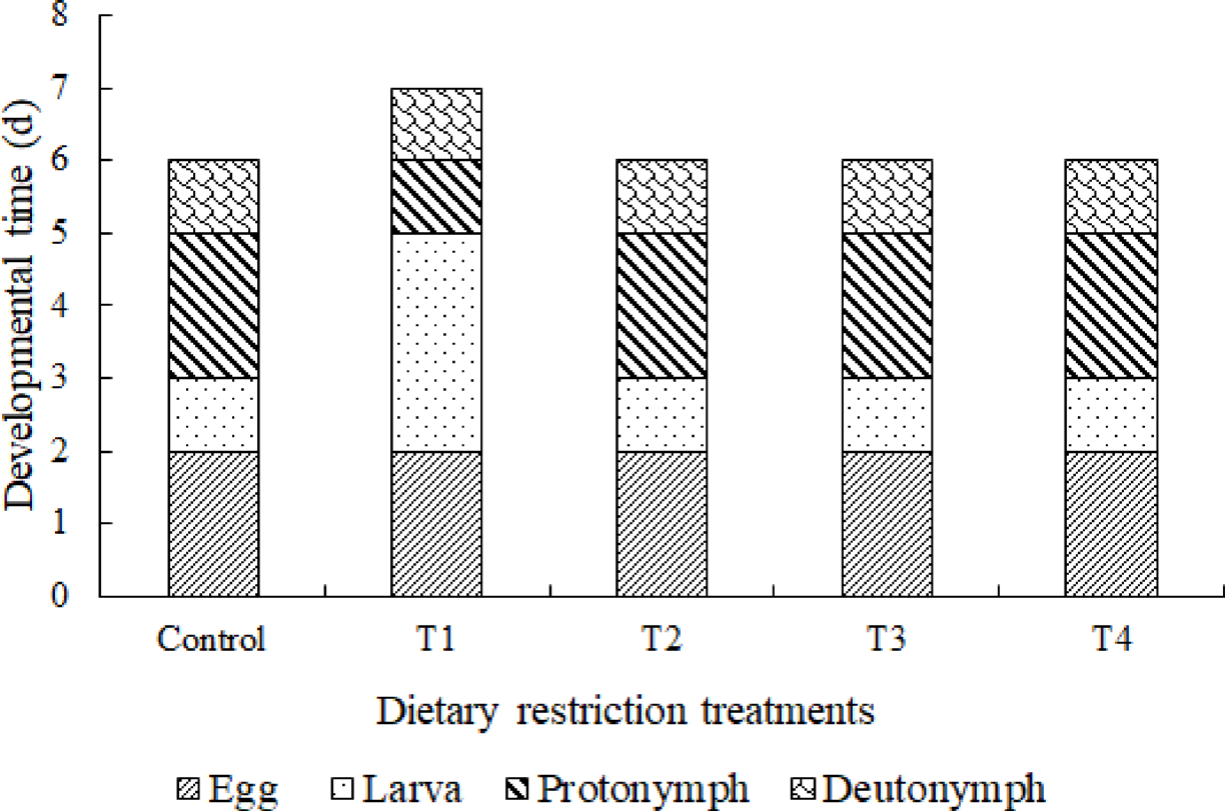
The developmental time (days) of egg, larva, protonymph (N1) and deutonymph (N2) of *Amblydromalus limonicus* under control and four dietary restriction treatment conditions, which were fasting for 1 day at the start of the larval (T1), protonymph (T2), deutonymph (T3) and adult (T4) stage.

One-day fasting during each developmental stage had no significant impact on immature survival (Table 1). However, fasting for one day during the larval stage extended the larval duration threefold (Fig. 1) and significantly increased its escape rate (Table 2).

**Table 1:** Survival rates (%) of immature of *Amblydromalus limonicus* under control and four treatment conditions, which were: Fasting for 1 day at the start of the larval (T1), protonymph (T2), deutonymph (T3) and adult (T4) stage.

	Egg	Larva	Protonymph	Deutonymph	Egg to adult
Control	100.0 (*n* = 50)	100.0 (*n* = 50)	98.0 ± 2.0 (*n* = 49)	91.8 ± 3.9 (*n* = 45)	90.0 ± 4.2 (*n* = 45)
T1	100.0 (*n* = 50)	92.0 ± 3.8 (*n* = 46)	84.8 ± 5.3 (*n* = 39)	87.2 ± 5.3 (*n* = 34)	68.0 ± 6.6 (*n* = 34)
T2	100.0 (*n* = 50)	98.0 ± 2.0 (*n* = 49)	93.9 ± 3.4 (*n* = 46)	84.8 ± 5.3 (*n* = 39)	78.0 ± 5.9 (*n* = 39)
T3	100.0 (*n* = 50)	96.0 ± 2.8 (*n* = 48)	91.7 ± 4.0 (*n* = 44)	90.9 ± 4.3 (*n* = 40)	80.0 ± 5.7 (*n* = 40)
T4	100.0 (*n* = 50)	98.0 ± 2.0 (*n* = 49)	95.9 ± 2.8 (*n* = 47)	87.2 ± 4.9 (*n* = 41)	82.0 ± 5.4 (*n* = 41)
χ^2^		5.94	7.50	1.55	7.74
df		4	4	4	4
*P*		0.20	0.11	0.82	0.10

**Table 2:** Escape rate (%) of *A. limonicus* under control and four treatment condition.

	Egg	larva	Protonymph	Deutonymph	Egg to adult
Control	0 (*n* = 50)	2.0 ± 2.0 (*n* = 49)	20.4 ± 5.8 (*n* = 39)	17.9 ± 6.1 (*n* = 32)	36.0 ± 6.8 (*n* = 32)
T1	0 (*n* = 50)	14.0 ± 4.9 (*n* = 43)	2.3 ± 2.3 (*n* = 42)	21.4 ± 6.3 (*n* = 33)	34.0 ± 6.7 (*n* = 33)
T2	0 (*n* = 50)	0 (*n* = 50)	12.0 ± 4.6 (*n* = 44)	20.5 ± 6.1 (*n* = 35)	30.0 ± 6.5 (*n* = 35)
T3	0 (*n* = 50)	2.0 ± 2.0 (*n* = 49)	18.4 ± 5.5 (*n* = 40)	17.5 ± 6.1 (*n* = 33)	34.0 ± 6.7 (*n* = 33)
T4	0 (*n* = 50)	0 (*n* = 50)	10.0 ± 4.2 (*n* = 45)	20.0 ± 6.0 (*n* = 36)	28.0 ± 6.3 (*n* = 36)
χ^2^		20.06	8.47	0.29	0.99
df		4	4	4	4
*P*		0.00	0.08	0.99	0.91

Four treatments: Fasting for one day at the start of larva (T1), protonymph (T2), deutonymph (T3) and adult stage (T4).

### Adult female reproductive parameters

The duration of the pre-oviposition period was very short (Table 3) and did not differ significantly among treatments (F = 0.546, *df* = 4, *P* = 0.703). Similarly, the week-long oviposition period showed no significant differences (F = 0.712, *df* = 4, *P* = 0.951). However, fasting of each stage for just one day significantly extended the duration of the post-oviposition period by 3–7 days on average (Table 3); larval fasting extended it (by 63%), which was significantly more than in other treatments (F = 77.726, *df* = 4, *P* < 0.001). Female daily reproductive rates and lifetime fecundities were not significantly different among the five groups (F = 0.173, *df* = 4, *P* = 0.951; F = 0.122, *df* = 4; *P* = 0.974; respectively) (Table 3).

**Table 3:** Effects of dietary restriction on reproductive lifespan and investment of female Am*blydromalus limonicus* under under control and four treatment conditions, which were: Fasting for 1 day at the start of the larval (T1), protonymph (T2), deutonymph (T3) and adult (T4) stage.

Parameters	Control	T1	T2	T3	T4	*P*
Pre-oviposition period (d)	1.3 ± 0.17a	1.7 ± 0.21a	1.4 ± 0.15a	1.3 ± 0.17a	1.3 ± 0.17a	0.70
Ovi-position period (d)	6.7 ± 0.37a	6.5 ± 0.43a	6.6 ± 0.31a	6.3 ± 0.24a	6.6 ± 0.29a	0.95
Post-oviposition period (d)	14.4 ± 0.34a	23.5 ± 0.43b	17.5 ± 0.31c	18.0 ± 0.29c	20.7 ± 0.44d	0.00
Daily reproductive rate (Eggs/d)	0.9 ± 0.02a	1.0 ± 0.03a	1.0 ± 0.03a	1.0 ± 0.02a	1.0 ± 0.02a	0.95
Life time fecundity (eggs/female)	6.2 ± 0.22a	6.2 ± 0.31a	6.3 ± 0.19a	6.1 ± 0.26a	6.3 ± 0.29a	0.97

Values are mean ± SE. Value within the same row followed by the different letters are significantly different (*P* < 0.05, Tukey’s test).

### Male and female lifespan

**Fig. 2:**
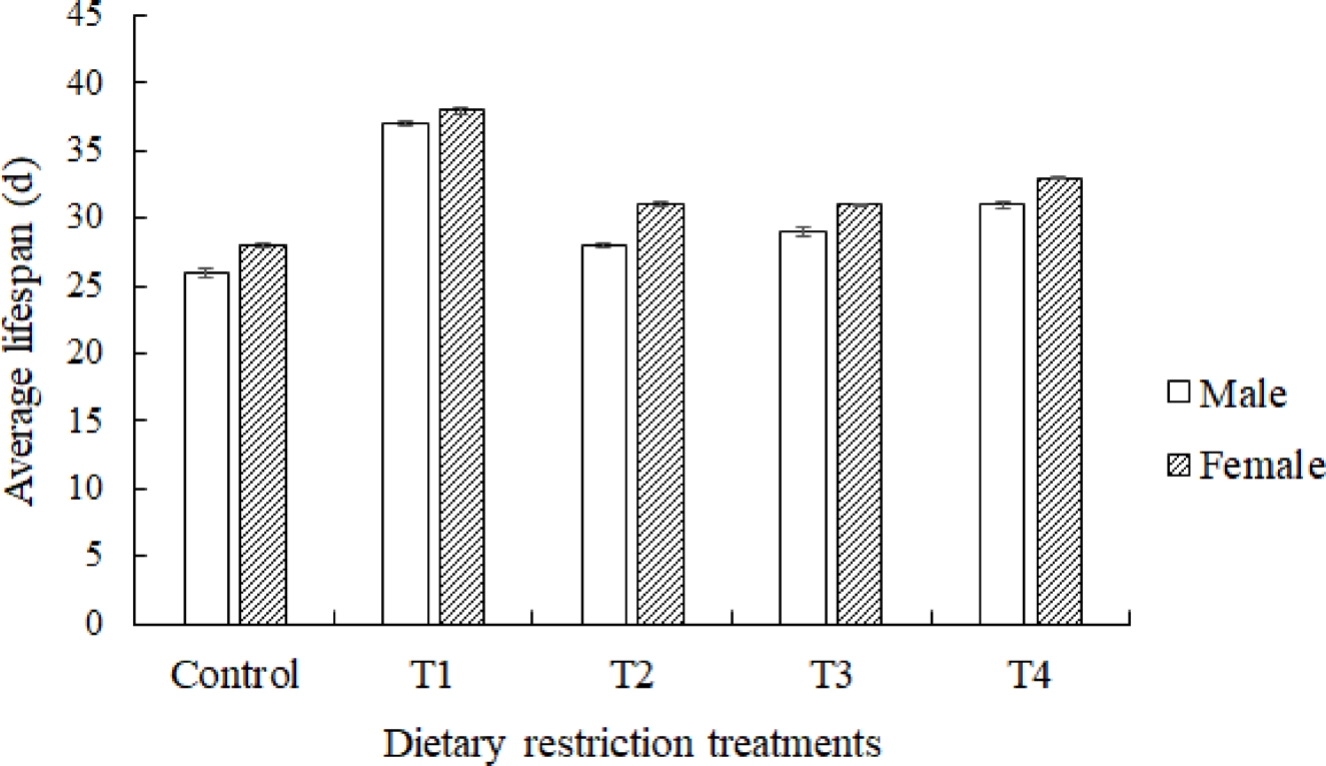
Average lifespan of male and female *Amblydromalus limonicus* under control and four dietary restriction treatment conditions, which were fasting for 1 day at the start of the larval (T1), protonymph (T2), deutonymph (T3) and adult (T4) stage.

The lifespans of female *A. limonicus* (F = 507.005; *df* = 4; *P* =  < 0.001) and male *A. limonicus* (F = 213.206; *df* = 4; *P* =  < 0.001) were the longest when larvae were fasting for 1 day (Fig. 2). In all treatments, the females outlived the males (F = 156.080; *df* = 1; *P* < 0.001), but there was no significant interaction between treatments and sex (F = 1.94; *df* = 1;* P* = 0.108).

### Relationship of body size (perimeter of dorsal shield) and longevity under short-term dietary restriction

**Fig. 3:**
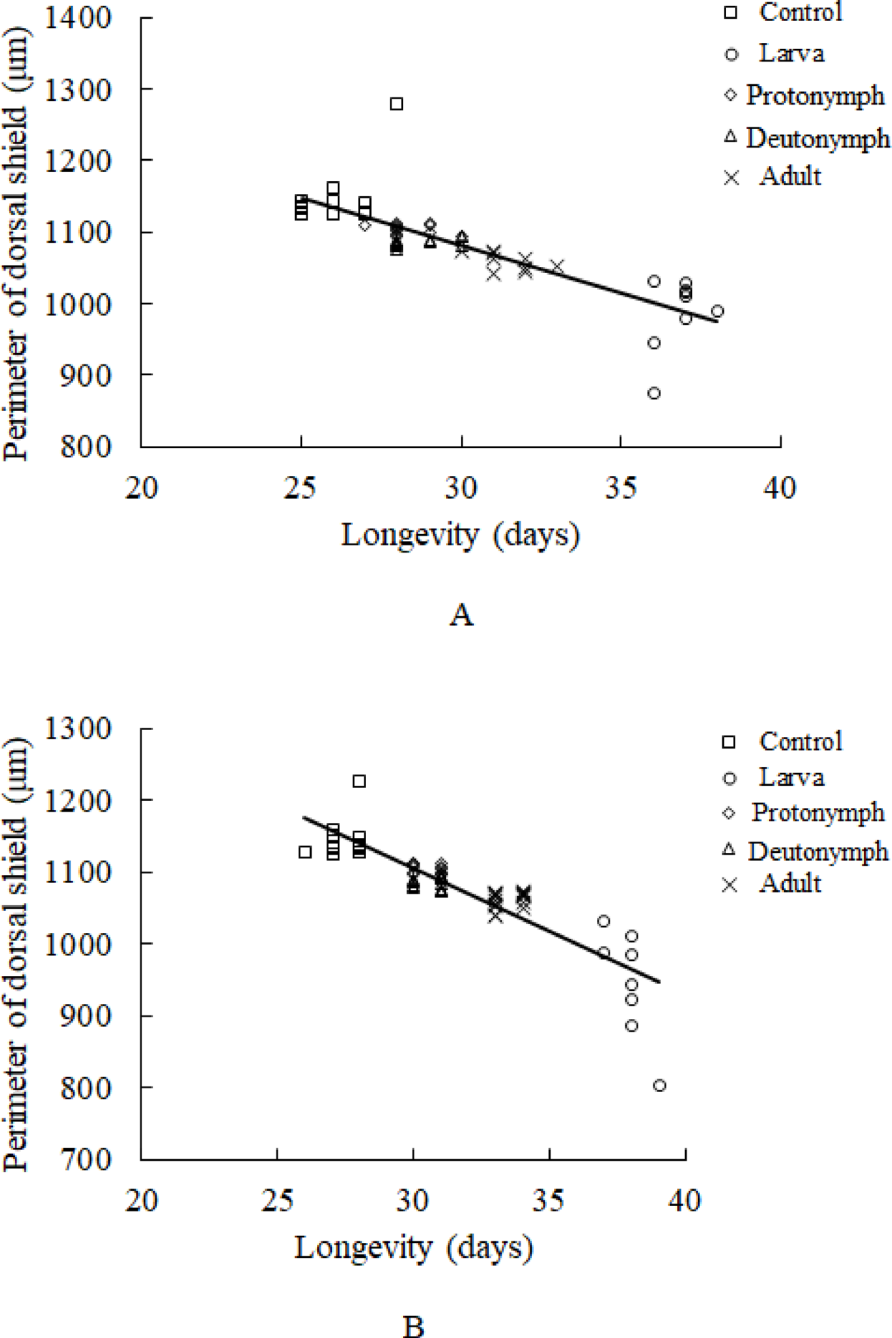
Body size and sex. (**A**) Correlations between the perimeter of dorsal shield and longevity of male *Amblydromalus limonicus* under dietary restriction. (**B**) Correlations between the perimeter of dorsal shield and lifespan of female *A. limonicus* under control conditions and dietary restriction for 1 day at the start of larva, protonymph (N1), deutonymph (N2) and adult stage.

We found significantly different perimeter shield values, relating to treatment (Table 4). The perimeter of the dorsal shield of control group was the longest, but the shortest for the T1 group –when each larva experienced fasting during its first day (F = 94.647; *df* = 4; *P* < 0.001) (Table 4). There were no significant differences in shield values between the sexes (Table 4). There was a significantly negative correlation between the perimeter of dorsal shield and lifespan, in both male (r = − 0.813, *P* < 0.001) and female (r = − 0.876, *P* < 0.001) mites (Fig. 3).

**Table 4:** Perimeter of dorsal shield of *Amblydromalus limonicus* under control and four dietary restriction treatment conditions, which were: Fasting for 1 day at the start of the larval (T1), protonymph (T2), deutonymph (T3) and adult (T4) stage.

	Control	T2	T3	T4	T1	*P*
Dorsal shield of female	1148.92 ± 13.39a	1103.98 ± 1.98c	1085.40 ± 1.76c	1061.79 ± 2.57c	987.88 ± 16.84b	*P* < 0.001
Dorsal shield of male	1142.67 ± 6.92a	1103.80 ± 1.90c	1084.30 ± 2.08c	1058.63 ± 3.93c	946.08 ± 26.42b	*P* < 0.001
*P*	*P* = 0.406	*P* = 0.404	*P* = 0.402	*P* = 0.409	*P* = 0.386	

Means ± SE within the same row followed by the different letters are significantly different (*P* < 0.05, Tukey’s test).

### Sex-specific response to short-term fasting

**Fig. 4:**
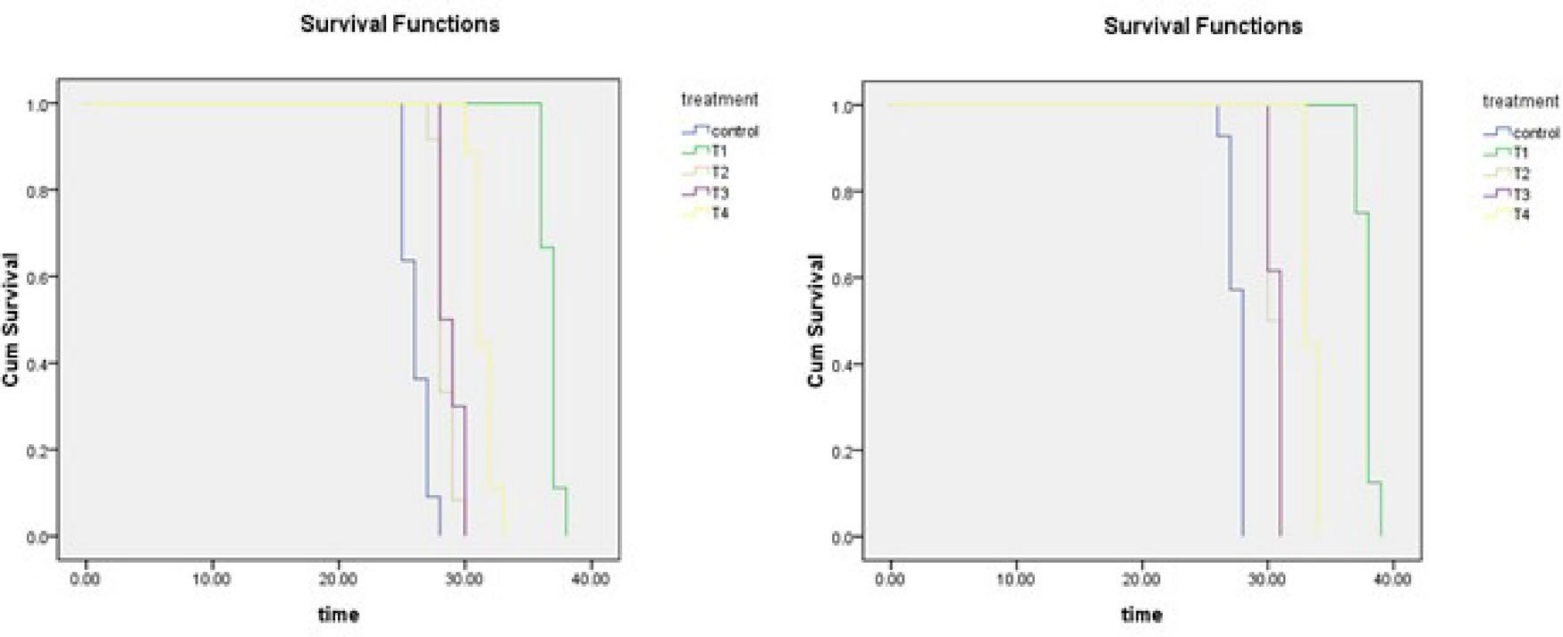
Survival functions. (**A**) Survivorship of male *Amblydromalus limonicus* subjected to dietary restriction under control conditions and four treatments; (**B**) Survivorship of female *A. limonicus* subjected to dietary restriction under control conditions and four treatments. The four dietary restriction treatment conditions were: Fasting for 1 day at the start of the larval (T1), protonymph (T2), deutonymph (T3) and adult (T4) stage.

The Kaplan–Meier estimate of the survivor function showed that *A. limonicus* had the shortest survival time in the control group; and longer survival time when larvae experience 1-day fasting both in male and female (Fig. 4). Comparisons between any two treatments of survival time for male and female *A. limonicus* are shown in Table 5. Except for the comparison between T2 and T3 which showed no significance (*P* = 1.191 for male and *P* = 0.596 for female), all other comparisons between any two treatments of survival time had significant differences (*P* < 0.001), in full agreement with general patterns in lifespan duration between treatments (Fig. 2).

**Table 5:** Comparisons between any two treatments of survival time of male and female *Amblydromalus limonicus* under control and four dietary restriction treatment conditions, which were fasting for 1 day at the start of the larval (T1), protonymph (T2), deutonymph (T3) and adult (T4) stage.

		Control	Control	T1	T1	T2	T2	T3	T3	T4	T4
Male	Control			χ^2^ = 19.937	*P* = 0.000	χ^2^ = 17.303	*P* = 0.000	χ^2^ = 17.456	*P* = 0.000	χ^2^ = 19.937	*P* = 0.000
	T1	χ^2^ = 19.937	*P* = 0.000			χ^2^ = 20.934	*P* = 0.000	χ^2^ = 18.806	*P* = 0.000	χ^2^ = 18.606	*P* = 0.000
	T2	χ^2^ = 17.303	*P* = 0.000	χ^2^ = 20.934	*P* = 0.000			χ^2^ = 1.713	*P* = 1.191	χ^2^ = 19.631	*P* = 0.000
	T3	χ^2^ = 17.456	*P* = 0.000	χ^2^ = 18.806	*P* = 0.000	χ^2^ = 1.713	*P* = 1.191			χ^2^ = 16.489	*P* = 0.000
	T4	χ^2^ = 19.937	*P* = 0.000	χ^2^ = 18.606	*P* = 0.000	χ^2^ = 19.631	*P* = 0.000	χ^2^ = 16.489	*P* = 0.000		
Female	Control			χ^2^ = 17.531	*P* = 0.000	χ^2^ = 23.469	*P* = 0.000	χ^2^ = 24.920	*P* = 0.000	χ^2^ = 32.068	*P* = 0.000
	T1	χ^2^ = 17.531	*P* = 0.000			χ^2^ = 17.286	*P* = 0.000	χ^2^ = 17.347	*P* = 0.000	χ^2^ = 20.610	*P* = 0.000
	T2	χ^2^ = 23.469	*P* = 0.000	χ^2^ = 17.286	*P* = 0.000			χ^2^ = 0.324	*P* = 0.569	χ^2^ = 31.663	*P* = 0.000
	T3	χ^2^ = 24.920	*P* = 0.000	χ^2^ = 17.347	*P* = 0.000	χ^2^ = 0.324	*P* = 0.569			χ^2^ = 31.226	*P* = 0.000
	T4	χ^2^ = 32.068	*P* = 0.000	χ^2^ = 20.610	*P* = 0.000	χ^2^ = 31.663	*P* = 0.000	χ^2^ = 31.226	*P* = 0.000		

Log Rank test was used in SPSS (*P* < 0.05).

## Discussion

Although DR has been shown to retard aging and extend lifespan in a wide range of species^7,8^, DR or IF were often applied to experimental animals over an extended period of their lives. Studies on the effects of very short-term DR (< 5% of lifespan, and only once) have been attempted rarely. In this study, we showed very interesting effects of STFEL on the performance of the predatory mite species *A. limonicus*, which has a total lifespan of about a month.

The impact of DR, particularly fasting at various frequencies, on lifespan has been a topic of longstanding debate. Although some studies with poorly designed methods have yielded surprising results, most well-controlled studies confirmed that DR and fasting can prolong mean and maximum lifespan in many organisms—including dogs, rodents, worms, flies, yeasts, and prokaryotes^28^. It has been assumed that extended lifespan of DR evolved as an adaptive strategy to overcome the period during which food is in shortage^29^. Researchers have also predicted that, relative to the natural lifespan of organisms, the extended lifespan would be much more prominent in short-lived species than in the long-lived species^30,31^. Our study with the short-lived arthropod *A. limonicus* agreed with this: We found the mite’s lifespan was prolonged by almost 10 days (i.e. 37.7% of their total lifespan) when the larvae were fasted for 1 day.

Similar findings have been reported in other mites, such as *Tetranychus urticae* Koch, 1836^5^, in which the female lifespan was prolonged by about 20% compared to individuals fed ad libitum*.* It should be noted that, in this study, the predatory mites were only starved for 1 day, which is different from some other studies where fasting lasted for a longer term. For example, in the study with *T. urticae*, the mites were subjected to IF throughout their adult stage. In comparison, our study highlighted that even short-term fasting in early life could have profound and long-lasting effects on the mites, and extend their lifespans in later life. Moreover, it is interesting that although starvation in different life stage all prolonged the lifespan of predatory mites in our current study, the most obvious effects was observed in mites which were fasted for 1 day in their larval stage. For *A. limonicus*, the larval stage is a facultative feeding stage—larvae can survive and develop to the next stage with or without feeding^32,33^. This study implied that plasticity in feeding during larval stage enabled the predatory mite to adapt much better when starvation occurred. It also further suggested that short-term DR in early life stages, and not necessarily long-term DR, is enough to induce beneficial effects in extending lifespan. This assumption is also supported by studies using the fruit fly as a model organism^34^, in which IF limited to middle life prolonged lifespan.

Some previous studies exploring the underlying mechanism of prolonged lifespan have pointed out that this benefit come at the cost of decreased lifetime fecundity, namely that there is a trade-off^35^. However, in our current study, the daily reproductive rate and lifetime fecundity did not differ across treatments. Additionally, no obvious changes in other reproductive parameters were found. The mites starved for 1 day all showed a similar pre-oviposition period and oviposition period. Only the post-reproductive duration differed among treatments, with the mites starved at the larval stage having the longest duration, and the control having the shortest—showing a similar trend to the total lifespan. These results indicated that the prolonged lifespan resulted mainly from the extend period in the post-reproductive stage. The conflict with Snell’s trade-off theory may be attributed to the fact that a post-reproductive lifespan is not prevalent and is rarely recorded in longevity studies^36^. Further studies with more attention to this life history trait are warranted to identify the relationship between longevity and post-reproductive lifespan.

In this study, we found that the body size of adult mites (as measured by the perimeter of their dorsal shield) differed significantly among treatments, being the longest in the control and shortest in the larval stage DR treatment, indicating starvation prolonged the larval stage and reduced the final body size of this predatory mite. Our study also found that the body size has a negative correlation with the longevity of predatory mites across treatments: The smaller mites lived longer than the bigger ones. These findings conflicted with those from a study with two-spotted spider mites, *Tetranychus urticae* Koch, in which no correlation was detected between body size and lifespan^37^. This divergence could result from the parameter for body size being different between these two studies: In Li and Zhang the body size were measured by the distance between the bases of paired *sce* setae on the prodorsal shield^37^; whereas we estimated body size by measuring the dorsal length of the shield because an increase in length of the dorsal shield does not always correspond with a proportional increase in dorsal shield width^38^. Therefore, we employed the perimeter of the dorsal shield suggested by Vangansbeke et al. in this study^39^. This indicator tends to be a better parameter to measure body size of mites to determine the influence of DR.

Our study indicates a positive correlation between fasting and escape rates in *A. limonicus*, likely reflecting its survival strategy under resource scarcity. Food deprivation may prompt migration to avoid competition or seek new resources, aligning with Vangansbeke et al. on cannibalism and Samaras et al. on pollen supplementation^40,41^. This highlights that maintaining stable food supplies (e.g., pollen or suitable prey) in biocontrol applications could reduce escape rates and improve efficacy.

The practical implications of these findings in our study for biological control programs can be emphasized as follows: (1) Optimizing mass-rearing protocols for predatory mites: The study shows that short-term fasting during the larval stage significantly extends lifespan. In large-scale production of *A. limonicus*, introducing a 1-day fasting period at the start of the larval stage (e.g., immediately after hatching) could be incorporated into rearing protocols to generate individuals with prolonged lifespans. Longer-lived predatory mites would maintain effective pest control over extended periods in the field, reducing the need for repeated releases and lowering associated costs while enhancing the sustainability of pest management^11,42^. (2) Targeting sensitive developmental stages for improved control efficiency: Given that the facultative-feeding larval stage is the most responsive to fasting-induced lifespan extension, biological control practices can focus on standardizing fasting interventions during this critical period. For example, by manipulating food availability at the larval stage in captive rearing, practitioners can select or enhance populations with enhanced longevity, ensuring that released mites exhibit prolonged survival and sustained predation pressure on target pests (e.g., spider mites, thrips), thus optimizing control efficacy^43,44^. (3) Cross-species applicability: The study highlights the potential of early-life dietary interventions to modulate lifespan in arthropods, a concept applicable to other biocontrol agents such as lady beetles, lacewings, and parasitoid wasps. Future research can investigate species-specific “sensitive periods” for dietary restriction, enabling the development of standardized fasting protocols to improve the field performance of diverse natural enemies. This approach promotes precision in biological control, reducing reliance on chemical pesticides and aligning with sustainable, environmentally friendly agricultural practices^45^. In conclusion, by investigating the impact of short-term DR on the lifespan of the predatory mite *A. limonicus*, we clarified that DR dramatically extended the lifespan of mites that experienced DR in their larval stage, extending their lifespan by up to 37.7%. Contrary to the trade-off theory^35^, there was no obvious cost in reproduction: The extended lifespan could be attributed mainly to a prolonged post-reproductive lifespan. A notable finding was the negative correlation between body size and longevity; mites starved during the larval stage were smaller and lived longer. This suggests that body size reduction may be an adaptive response to DR, promoting longevity. These results highlight the profound and lasting effects of short-term DR at specific life stages, without compromising reproductive success. This study has enhanced our understanding of DR-induced lifespan extension and emphasizes the importance of considering life stage and body size in aging research, for a range of species. More studies on short-term DR during different life stages using a wider range of taxa are needed to fully understand their effects on aging and lifespan.

## Materials and methods

### Mite cultures

The laboratory culture of *A. limonicus* used in this paper originated from mites found on greenhouse capsicum leaves in 2012 in South Auckland, New Zealand, and was maintained in a cabinet set at 25 ± 1 °C, 85 ± 5% RH and a 16:8 h light: dark (L: D) photoperiod at Manaaki Whenua—Landcare Research, Auckland, New Zealand. Predatory mites were reared on a black plastic arena (7 × 7 cm) on a wet sponge in a plastic tray containing water to prevent mites from escaping and to provide access to water^46,47^. Several 3–4 cm pieces of black sewing thread dusted with fresh pollen of oriental cattail–*Typha orientalis* C. Presl were placed around the margin of the rearing arenas to serve as feeding stations and oviposition substrate. These black sewing threads were covered with some squares of small white cloth of similar sizes with fresh *T. orientalis* pollen to fed *A. limonicus.* Fresh pollen of *T. orientalis* was collected from a reserve in St Johns, Auckland. It was dried in an oven at 35 °C for 2 d and then stored at − 18 °C for the experiment^48^. Fresh pollen, dusted threads and white cloth squares were added to the rearing unit every other day, and mouldy ones were removed when they were detected.

### Methods and experiment

A modified Munger cell (upper diameter 6 mm, lower diameter 3 mm, height 3 mm), as described in detail by Liu and Zhang was used to rear individual mites in this experiment^24^. Water was provided to the mites by plugging a piece of tissue paper wick soaked in tap water through a hole in the bottom of the cell. To prepare cohorts of *A. limonicus* eggs (all 1-d old), groups of 10–20 young gravid females of *A. limonicus* were transferred to new black plastic arenas with pollen-dusted pieces of black sewing thread and white cloth. Eggs laid within the same 24 h were then transferred individually to these Munger cells. In total, 250 of these Munger cells, each containing 1 mite egg were prepared and assigned to either a control group, or to one of four treatment groups. Each treatment group involved fasting for 1 day at the start of a different feeding stage e.g. (i) larva—T1, (ii) protonymph—T2, (iii) deutonymph—T3, and (iv) adult—T4. There were 50 replicates per treatment.

In the control, mites were fed every day without fasting throughout their lifespan. Treatment 1 (T1) was for larval fasting (mites were fed pollen every day except the first day of the larval stage). Treatment 2 (T2) was for protonymph fasting (mites were fed pollen every day except the first day of the protonymph stage). Treatment 3 (T3) was for deutonymph fasting (mites were fed pollen every day except the first day of the deutonymphal stage). Treatment 4 (T4) was for adult fasting (mites were fed pollen every day except the first day of the adult stage). During the experiment, the survival of each mite was checked every 24 h, until all mites were dead. The number of mites escaped from the cell was also recorded and the escape rate was calculated as the proportion of mites that escaped from the cell in each treatment. The number of eggs laid by each female was also recorded each day. For all treatments and control, pieces of black sewing thread dusted with fresh pollen were added every other day until the adult mite died. Then all mites were mounted in Hoyer’s medium and dried in an oven 45 °C for 1 wk.

### Measurement method

All specimens were examined, measured, and photographed with a Nikon eclipse Ni 90 microscope. All measurements are provided in micrometers. The measuring method follows Ma et al., except in the larval stage, the dorsal length was measured from j1 to the middle point of Z4 to Z4 (due to the fact that the larva does not have a single shield covering the whole idiosoma). Each measurement shows the average (minimum–maximum)^49^.

### Statistical analysis

The survival time of *A. limonicus* (using the Kaplan–Meier estimate of the survivor function) were analyzed in Genstat® 18 statistics software (VSN International Limited, https://vsni.co.uk/software/genstat/). Other analyses were carried out using SPSS® 27.0 statistics software (SPSS Inc., Chicago, IL, USA). One-way ANOVAs and multiple comparisons and Tukey tests were used to analyze the female reproductive parameters, including: The pre-oviposition period, oviposition period, post-oviposition period, daily reproductive rate, and life time fecundity; all of these parameters were compared with dietary regimes as the main factor. The distribution of data for each parameter was checked to find whether they met the assumption of normal distribution before analysis. Log Rank test was used to compare between any two treatments of survival time of male (or female) *A. limonicus* under control and four dietary restriction treatment conditions.

## Data Availability

All data generated during this study are included in this published article.
